# The epidemiology and socioeconomic associates of adverse effects of medical treatment in the Eastern Mediterranean Region from 1990 to 2021

**DOI:** 10.1038/s41598-025-18289-z

**Published:** 2025-09-26

**Authors:** Azin Kadkhodamanesh, Paria Dehesh, Bita Mesgarpour, Seyed Aria Nejadghaderi, Hamid Sharifi

**Affiliations:** 1 HIV/STI Surveillance Research Center, and WHO Collaborating Center for HIV Surveillance, Institute for Futures Studies in Health, Kerman University of Medical Sciences, Kerman, Iran; 2 Social Determinants of Health Research Center, Institute for Futures Studies in Health, Kerman University of Medical Sciences, Kerman, Iran; 3 Department of Biostatistics and Epidemiology, School of Public Health, Kerman University of Medical Sciences, Kerman, Iran; 4 Research and Technology Department, National Institute for Medical Research and Development (NIMAD), Tehran, Iran; 5 Knowledge Hub for Migrant and Refugee Health, Institute for Futures Studies in Health, Kerman University of Medical Sciences, Kerman, Iran; 6 Institute for Global Health Sciences, University of California, San Francisco, San Francisco, CA USA

**Keywords:** Adverse effects of medical treatment, Medical errors, Patient safety, Incidence, Disability-a­djusted life years, Death, Global burden of disease, Drug safety, Epidemiology

## Abstract

Concerns about the adverse effects of medical treatment (AEMT) are increasing along with the global use of medical treatments. However, comprehensive information regarding the epidemiology of AEMT in the Eastern Mediterranean Region (EMR) is lacking. This study assessed the burden of AEMT in the EMR from 1990 to 2021 by age, sex, and socio-demographic index (SDI). Data on the prevalence, incidence, deaths, and disability-adjusted life years (DALY) of AEMT in the EMR was extracted from the Global Burden of Disease 2021 study. Counts, age-standardized rates, percent changes in the rates, and 95% uncertainty intervals were reported. In the EMR, the burden of AEMT declined over the past three decades, with notable variation across countries and population groups. In 2021, females experienced a higher burden than males in most EMR countries and at the regional level. The highest DALY and death rates occurred in early childhood and among the elderly. Countries with a high SDI tended to have higher incidence rates, whereas those with a lower SDI experienced higher DALY rates. The epidemiology of AEMT exhibited considerable variation based on age, sex, and socioeconomic status in the EMR, highlighting the need for context-specific patient safety initiatives and medical error prevention programs across the region.

**Supplementary Information:**

The online version contains supplementary material available at 10.1038/s41598-025-18289-z.

## Introduction

Adverse effects of medical treatment (AEMT) refer to any disease, injury, or complication that develops due to medical care and results in suffering, prolonged recovery period, diminished quality of life, or even death. Postoperative infections, drug hypersensitivity reactions, and inappropriate medication prescriptions are some examples of AEMT^1^. The global population increased by 44.6% between 1990 and 2019, while the incidence cases of AEMT grew even more rapidly, rising by 59.3%^2^. This trend prompted increased efforts to gather information, identify underlying causes, and implement monitoring systems, including methods for patient safety and medical malpractice reporting^3^.

Despite global improvements, AEMT remains a major public health issue, particularly in underdeveloped countries. All World Health Organization (WHO) regions exhibited a downward trend in disability-adjusted life years (DALY) due to AEMT. In the Eastern Mediterranean Region (EMR), DALY rates decreased by 23.2% from 1990 to 2017; however, the burden remains higher in the EMR than in most other WHO regions. Patient safety and healthcare quality in the EMR face unique challenges due to extreme adversity settings. Key issues include financing problems, service inaccessibility, insecurity of health workers, breakdowns in health systems, and inadequate infrastructure^4^. Notably, while years of life lost (YLL) rates due to AEMT fell from 109.3 to 83.2 per 100,000 population, years lived with disability (YLD) increased from 2.1 to 2.4 per 100,000 population, indicating a shift in the burden from mortality to long-term morbidity^5^. Consequently, the WHO has implemented a plan to reduce occurrences of AEMT by 2022^6^. Approximately 8.2% of hospital admissions in developing countries, particularly in the EMR and African regions, experience at least one adverse event, 83% of which are preventable^7^. Medical errors have a wide variety of effects on patients, families, and communities, ranging from fatal outcomes to lifetime impairment and decreased quality of life^8^. Therefore, to fully assess the burden of AEMT, it is essential to consider disability and associated costs and mortality^9^.

Previous studies have primarily focused on examining the burden of AEMT at the global level^3,5,10^ and in specific countries such as India^11^ and the United States^12^. Furthermore, some studies have specifically targeted certain groups, such as older adults in the United States from 1990 to 2019^13^ or children in the United States from 2000 to 2019^14^. These studies evaluated indicators such as DALY, YLD, and YLL using earlier iterations of Global Burden of Disease (GBD) data. However, to the best of our knowledge, no studies have been conducted on the disease burden and trends of AEMT in the EMR using recent data.

The EMR comprises 22 countries with diverse healthcare systems, economic conditions, and population health profiles^15^. In the past three decades, the region has witnessed significant improvements in healthcare systems due to investments in medical infrastructure, increased access to treatment, and the adoption of new medical technologies. However, these developments may also pose challenges to the safety and quality of medical care, potentially increasing the risk of AEMT. Using data from the GBD 2021 project, this study aimed to examine the epidemiology of AEMT in the EMR from 1990 to 2021 by age, sex, and its association with socio-demographic index (SDI). This research contributes to the growing body of knowledge on patient safety and healthcare quality, providing policymakers and healthcare professionals with valuable insights to inform targeted interventions and improve healthcare outcomes in the EMR.

## Methods

### Overview

The Global Study on Diseases, Injuries, and Risk Factors was created to estimate health losses resulting from 371 diseases and injuries, as well as 88 risk factors between 1990 and 2021, based on age, sex, and geographic location^16^. Compared with previous iterations of GBD (e.g., GBD 2019), the GBD 2021 research included several key improvements. First, more precise age groupings for children under five were added to the cause-of-death data. Second, improved statistical techniques were used better to quantify the impact of fewer frequent causes of death and to account for uncertainty in cause-of-death data. Third, a substantial amount of new data, such as verbal autopsy, surveillance, vital registry, and other data sources, were added to the study. Finally, the impact of COVID-19 and other pandemic-related mortality was investigated^16,17^.

### Data sources and definition

The Institute for Health Metrics and Evaluation developed the Global Health Data Exchange, a platform for the GBD dataset. The data is available at https://vizhub.healthdata.org/gbd-results/. We extracted data on the incidence, prevalence, DALY, and deaths of AEMT in the EMR and its 22 countries from 1990 to 2021. Moreover, percent changes in age-standardized rates were also extracted. Data was included from several sources, including surveys, as well as medical, hospital, emergency department, and insurance records. AEMT was defined based on the International Classification of Diseases (ICD). Table S1 represents the relevant ICD-9 and ICD-10 codes used for the definition of AEMT^17^.

### Data processing and modelling

The DisMod-MR 2.1 tool, a Bayesian meta-regression analysis of data on non-fatal health outcomes, was used for modelling. This model estimates incidence, prevalence, remission, and mortality even in locations with sparse or missing data by borrowing information from other countries in the same region and incorporating location-specific covariates. It ensures internally consistent and comparable estimates across different geographic and demographic settings.

However, the accuracy of these modelled estimates depends heavily on the availability and quality of primary input data. According to the GBD 2021 Data Input Sources Tool (https://ghdx.healthdata.org/gbd-2021/sources), no primary data on AEMT were available for nine EMR countries, Afghanistan, Djibouti, Morocco, Libya, Pakistan, Somalia, Sudan, Tunisia, and Yemen, during the 1990–2021 period. For Iran, Jordan, and Qatar, primary data were available but limited to selected years. The remaining countries had partial data availability, often restricted to data related to death or specific time points. These limitations indicate that many of the AEMT estimates for the EMR were derived primarily through modelling rather than empirical observation.

Uncertainty from data gaps, adjustments, and modelling assumptions is quantified through 95% uncertainty intervals (UIs) provided with all final estimates. However, estimates from low-data settings may still reflect higher uncertainty, which is acknowledged as a limitation of the modelling approach. YLL was calculated by deducting a deceased person’s age from their area’s average life expectancy. YLD was estimated by multiplying the prevalence by the associated disability weight. Finally, DALY was calculated by summing YLL and YLD to indicate the burden of the AEMT^16^.

The AEMT mortality rate per 100,000 people was calculated by dividing the number of AEMT-related deaths in a given year by the population of the same region. To address the influence of population structural characteristics (such as age and sex) on death rates, age-standardized death rates were calculated based on the GBD standard population.

### Statistical analysis

The 95% UI was calculated to account for the estimations’ uncertainty. Each step of the estimation process generated 500 iterations, and the 95% UIs were defined as the 25th and 975th values from the numerically ordered iterations. The final estimates were based on the mean values from these 500 iterations^16^.

Smoothing spline models were used to analyze the relationship between the SDI and the age-standardized incidence and DALY rates of AEMT. These outcomes are continuous variables derived by dividing event counts by population estimates and adjusting for age structure. Generalized additive models (GAMs) with penalized smoothing splines were used to flexibly model potential nonlinear associations between SDI and the incidence and DALY rates. To reflect uncertainty, we added confidence bands around the spline curves. To assess the robustness of the results, we performed sensitivity analyses by varying the smoothing parameter and comparing effect estimates across different SDI ranges. These effect estimates represent the predicted differences in incidence or DALY rates between selected SDI values based on the fitted models. The SDI considers three components: the average education level of people over 15, income per capita adjusted for inequality, and the total fertility rate of women under 25. Its value ranges from zero (less developed) to one (highly developed)^18^.

We reported the counts, age-standardized rates, and percent changes in the age-standardized rates, along with their 95% UIs, for AEMT from 1990 to 2021 worldwide, in the EMR, and in 22 countries of the EMR. We also reported the epidemiology of AEMT by sex and in different age groups with 5-year intervals. We used R (version 4.2.1) for all statistical analysis and visualization.

## Results

### Epidemiology of AEMT at the global, regional, and country levels

**Fig. 1:**
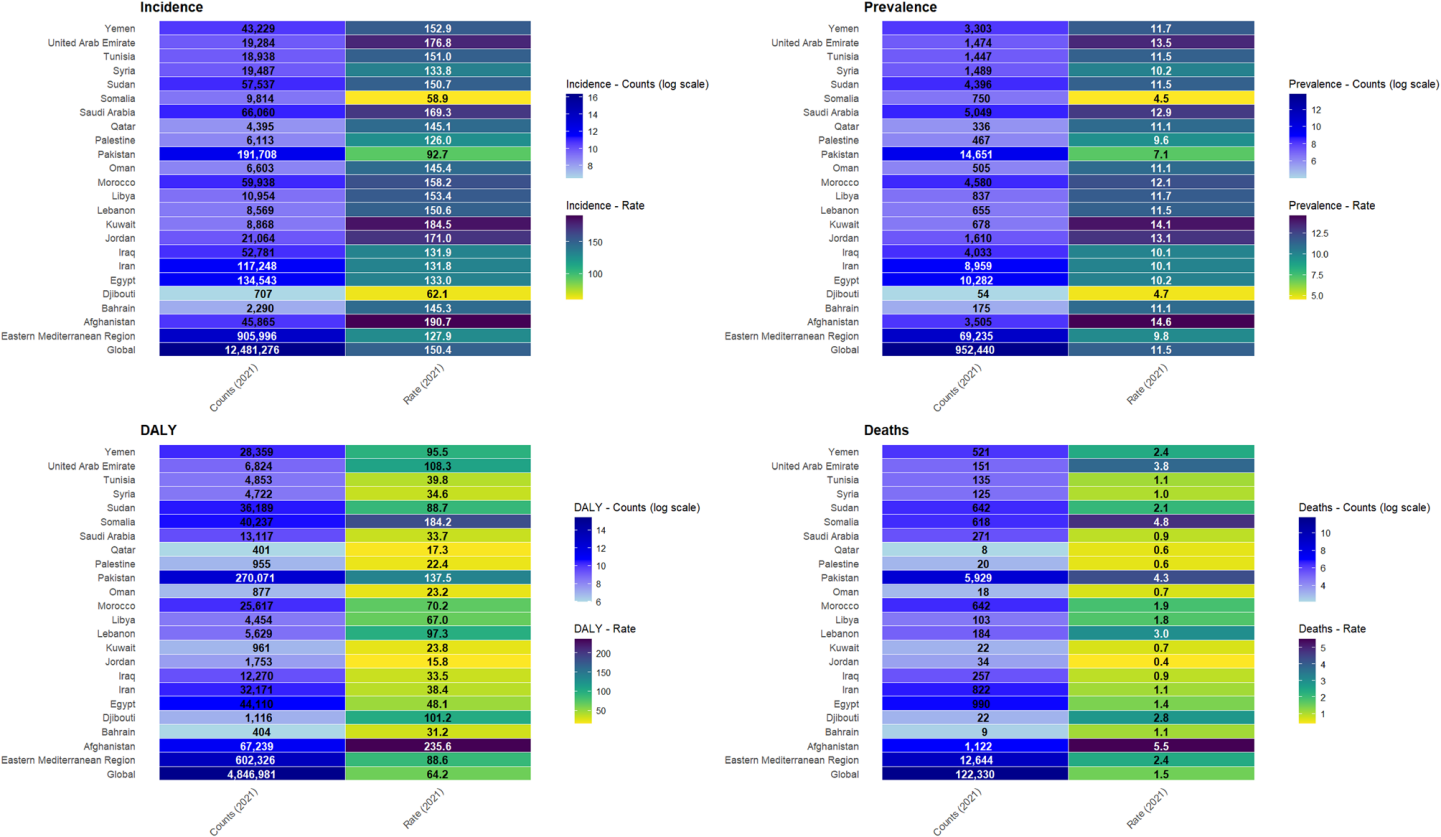
Heat maps illustrate prevalent cases, incident cases, deaths, and disability-adjusted life years (DALYs), along with their age-standardized rates per 100,000 population, due to adverse effects of medical treatments (AEMT) in 2021 for both sexes in the Eastern Mediterranean Region (EMR). (Generated from data available from http://ghdx.healthdata.org/gbd-results-tool).

In 2021, the age-standardized incidence rate of AEMT in the EMR was 127.9 (95% UI: 110.4, 149.8) per 100,000 population, reflecting an overall decrease of -18.7% (95% UI: -20.5, -17.0) since 1990. The global age-standardized incidence rate was higher at 150.4 (95% UI: 131.2, 171.8) per 100,000 population, with a smaller decrease of -5.3% (95% UI: – 7.9, -2.6) over the same period. The age-standardized point prevalence of AEMT in 2021 was 9.8 (95% UI: 7.7, 12.1) in the EMR and 11.5 (95% UI: 8.9, 14.1) per 100,000 globally. The age-standardized point prevalence of AEMT declined from 1990 to 2021 in both the EMR (-18.7% [95% UI: -20.5, – 17.0]) and globally (-5.3% [95% UI: -7.8, -2.7]), with the largest percentage change occurring in the EMR. In 2021, the global age-standardized DALY rate due to AEMT was 64.2 (95% UI: 51.1, 73.1) per 100,000 population, showing a -39.7% (95% UI: – 48.9, -31.2) decrease from 1990 to 2021. The age-standardized DALY rate in the EMR for 2021 was 88.6 (95% UI: 73.6, 103.2) per 100,000, with a – 46.3% (95% UI: -55.4, -35.4) decrease over the same period. The global and EMR age-standardized death rates of AEMT in 2021 were 1.5 (95% UI: 1.3, 1.7) and 2.4 (95% UI: 2.0, 2.8) per 100,000 population, respectively. Between 1990 and 2021, the percentage change in death rates decreased by -36.1% (95% UI: -43.7, -28.0) globally and by -40.7% (95% UI: -54.8, -27.3) in the EMR (Fig. 1 and Table S2).

Across the EMR countries in 2021, Afghanistan (190.7 [95% UI: 169.5, 217.4] per 100,000 population) and Somalia (58.9 [95% UI: 50.9, 67.8] per 100,000 population) recorded the highest and lowest age-standardized incidence rates of AEMT, respectively. All EMR countries exhibited a decrease in age-standardized incidence rates from 1990 to 2021, except for Pakistan, which showed an increasing pattern (11.8% [95% UI: 6.0, 16.9]). The most notable decreases were observed in Iran (-42.7% [95% UI: -44.5, -41.0]), while Somalia had the smallest percentage change (-6.6% [95% UI: -10.9, -1.4]). Similarly, Afghanistan (14.6 [95% UI: 11.4, 17.6]) and Somalia (4.5 [95% UI: 3.5, 5.5]) had the highest and lowest age-standardized prevalence of AEMT among the EMR countries, respectively. All countries in the region showed an overall negative trend in age-standardized prevalence from 1990 to 2021, except Pakistan (11.8% [95% UI: 5.8, 17.2]), the only country to report a positive percentage change. Iran (-42.7% [95% UI: -44.5, -41.0]) experienced the largest overall decrease, while Somalia (-6.6% [95% UI: -10.9, -1.4]) had the smallest decrease over this period. In 2021, the age-standardized DALY rate across the EMR countries ranged from the highest in Afghanistan (235.6 [95% UI: 160.3, 326.8] per 100,000 population) to the lowest in Jordan (15.8 [95% UI: 12.8, 19.2] per 100,000 population). Similarly, the highest and lowest age-standardized death rates in 2021 were observed in Afghanistan (5.5 [95% UI: 3.7, 7.8] per 100,000 population) and Jordan (0.4 [95% UI: 0.3, 0.5] per 100,000 population), respectively. From 1990 to 2021, all EMR countries showed a decreasing trend in both DALY and death rates due to AEMT. The greatest decreases in age-standardized DALY and death rates during this period were in Kuwait (-83.1% [95% UI: -85.9, -80.0] and − 80.9% [95% UI: -84.7, -76.9], respectively). In contrast, the smallest reductions in age-standardized DALY rates were observed in Somalia, with a change of -27.2% [95% UI: -50.1, 2.6], which was not statistically significant. Pakistan experienced a similar reduction of -27.5% (95% UI: -46.8, -7.4) in age-standardized DALY rates. Furthermore, Somalia recorded the smallest decrease in the age-standardized death rate at -17.2% (95% UI: – 40.4, 6.2), which also was not statistically significant (Fig. 1, Table S2, and Figure S1).

**Fig. 2:**
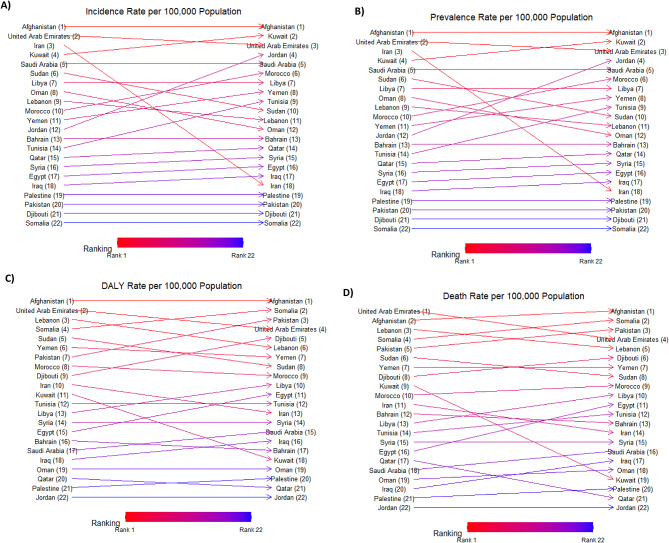
Ranking of age-standardized rates for incidence (**A**), prevalence (**B**), DALY (**C**), and death (**D**) of adverse effects of medical treatment (AEMT) per 100,000 population for both sexes in 1990 and 2021 in the Eastern Mediterranean Region (EMR), by country. DALY: disability-adjusted life year. (Generated from data available from http://ghdx.healthdata.org/gbd-results-tool).

In 1990, Afghanistan, the United Arab Emirates (UAE), and Iran had the highest age-standardized incidence rate and point prevalence of AEMT among both sexes in the EMR. In 2021, Afghanistan and the UAE remained among the top three countries with the region’s highest incidence and prevalence rates. Afghanistan, the UAE, and Lebanon were the countries with the highest age-standardized DALY and death rates in 1990. By 2021, Afghanistan still had the highest estimated age-standardized DALY and death rates due to AEMT among the EMR countries (Table S2, Fig. 2, and Figure S1).

**Fig. 3:**
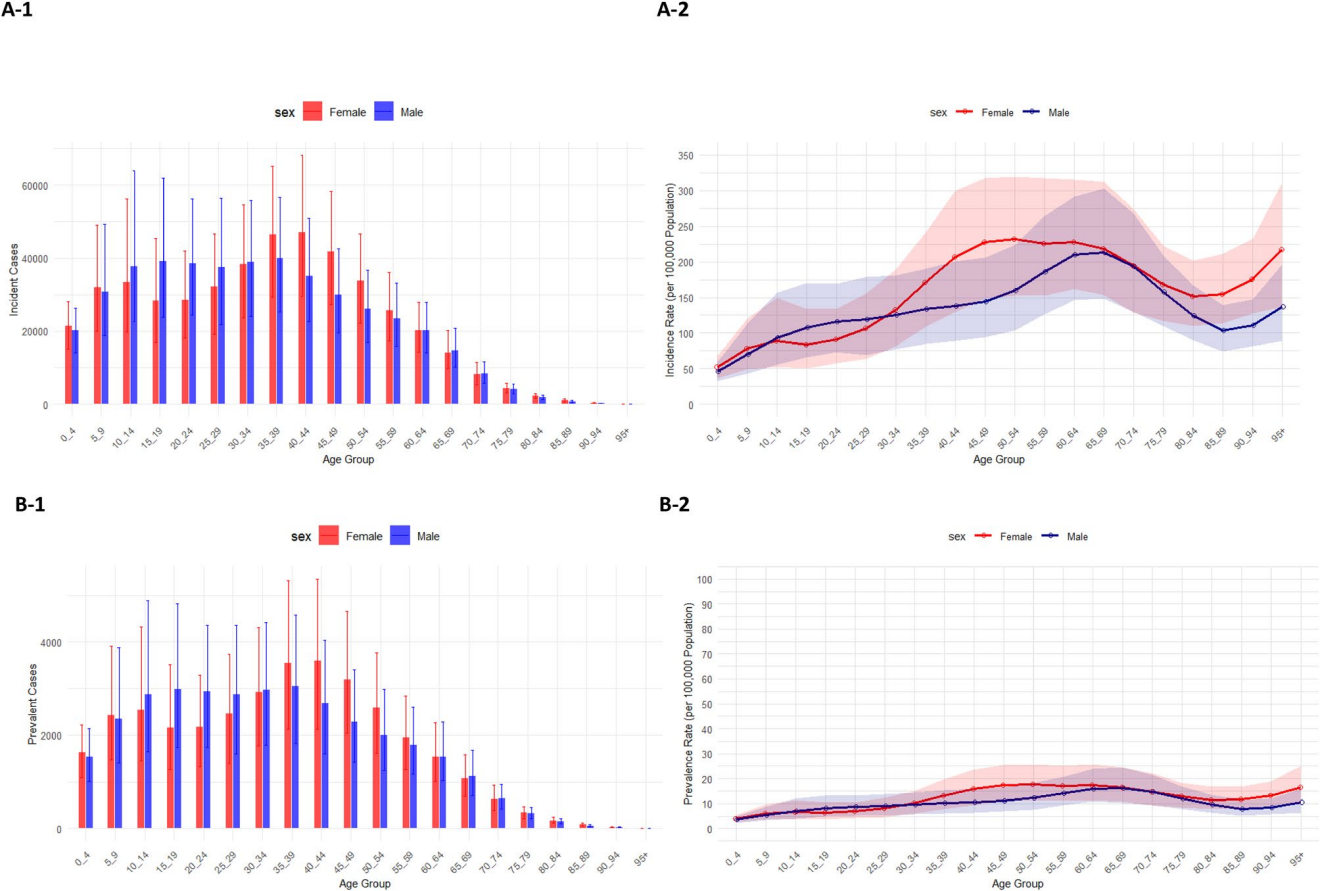

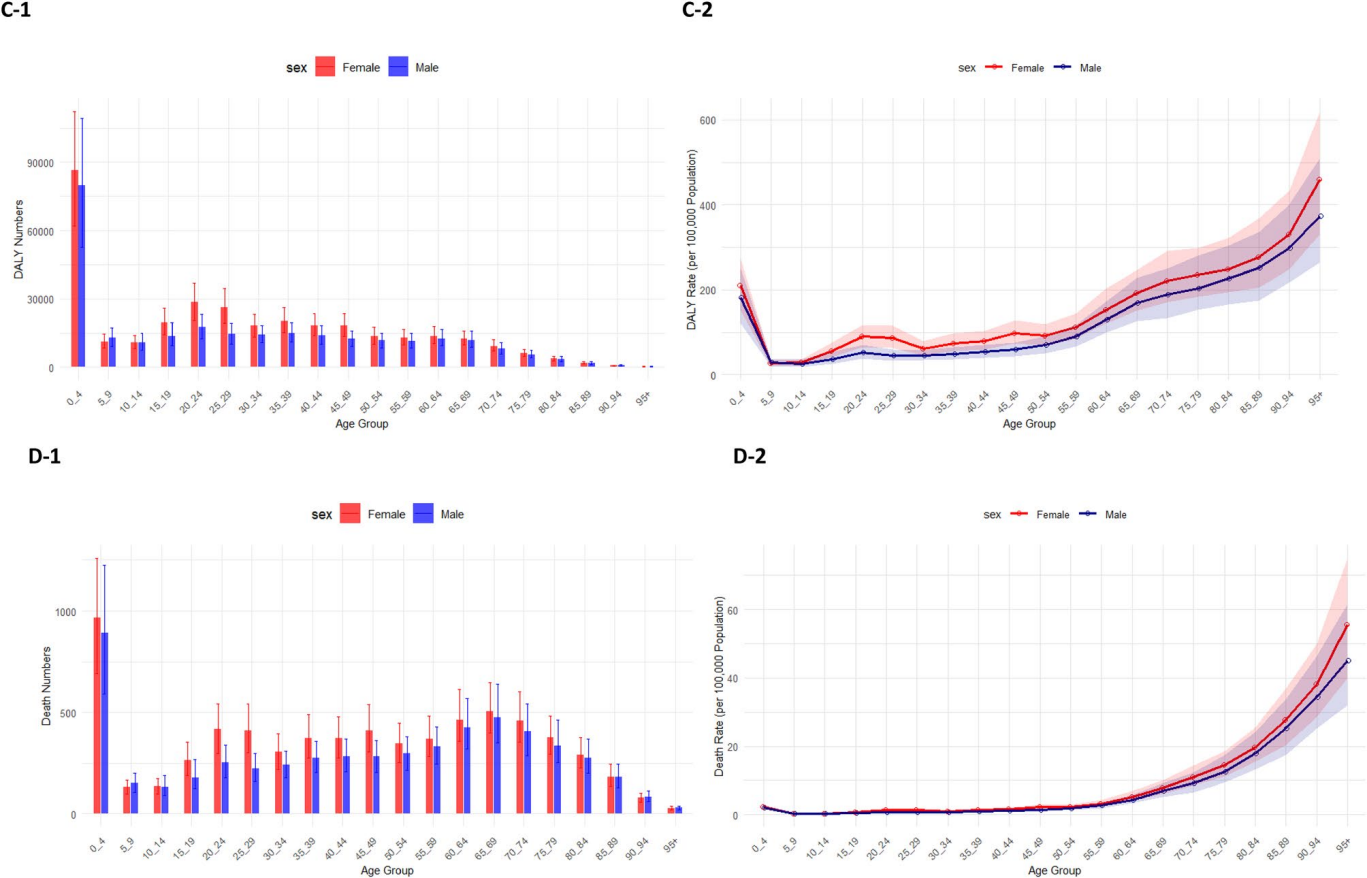
Number of incident cases (**A1**) and incidence rate (**A2**), number of prevalent cases (**B1**) and prevalence rate (**B2**), number of DALYs (**C1**) and DALY rate (**C2**), and the number of deaths (**D1**) and death rate (**D2**) for adverse effects of medical treatment (AEMT) (per 100,000 population) in the Eastern Mediterranean Region (EMR) in 2021, by age and sex. Error bars on the bar plots and shaded areas on the line plots represent 95% uncertainty intervals for numbers and rates, respectively. DALY: disability-adjusted life year. (Generated from data available from http://ghdx.healthdata.org/gbd-results-tool).

### Epidemiology of AEMT by age and sex

In 2021, incident and prevalent cases of AEMT among females in the EMR were highest in the age groups of 35 to 44. Among males, the highest number of incident and prevalent cases occurred between ages 10 and 39, with the highest being in the 35–39 age group (Fig. 3A1 and Fig. 3B1). Incidence and prevalence rates increased with age in both males and females, the highest at 50–54 years in females and 65–69 years in males for incidence, and at 45–69 years in females and 60–69 years in males for prevalence, followed by a slight decline in older age groups (Fig. 3A2 and Fig. 3B2).

The highest number of DALYs and deaths due to AEMT in the EMR was observed in early childhood (ages 0–4) for both males and females (Fig. 3C1 and Fig. 3D1). After the age group of 0–4 years, DALY and death rates decreased among those aged 5–9 years. This was followed by a slight increase in rates that plateaued until mid-age, then rose again, being the largest in the 95 + age group (Fig. 3C2 and Fig. 3D2). Across most age groups, females had slightly higher numbers of DALYs and deaths due to AEMT than males, except in the 5-9-year age group (Fig. 3C1 and Fig. 3D1).

**Fig. 4:**
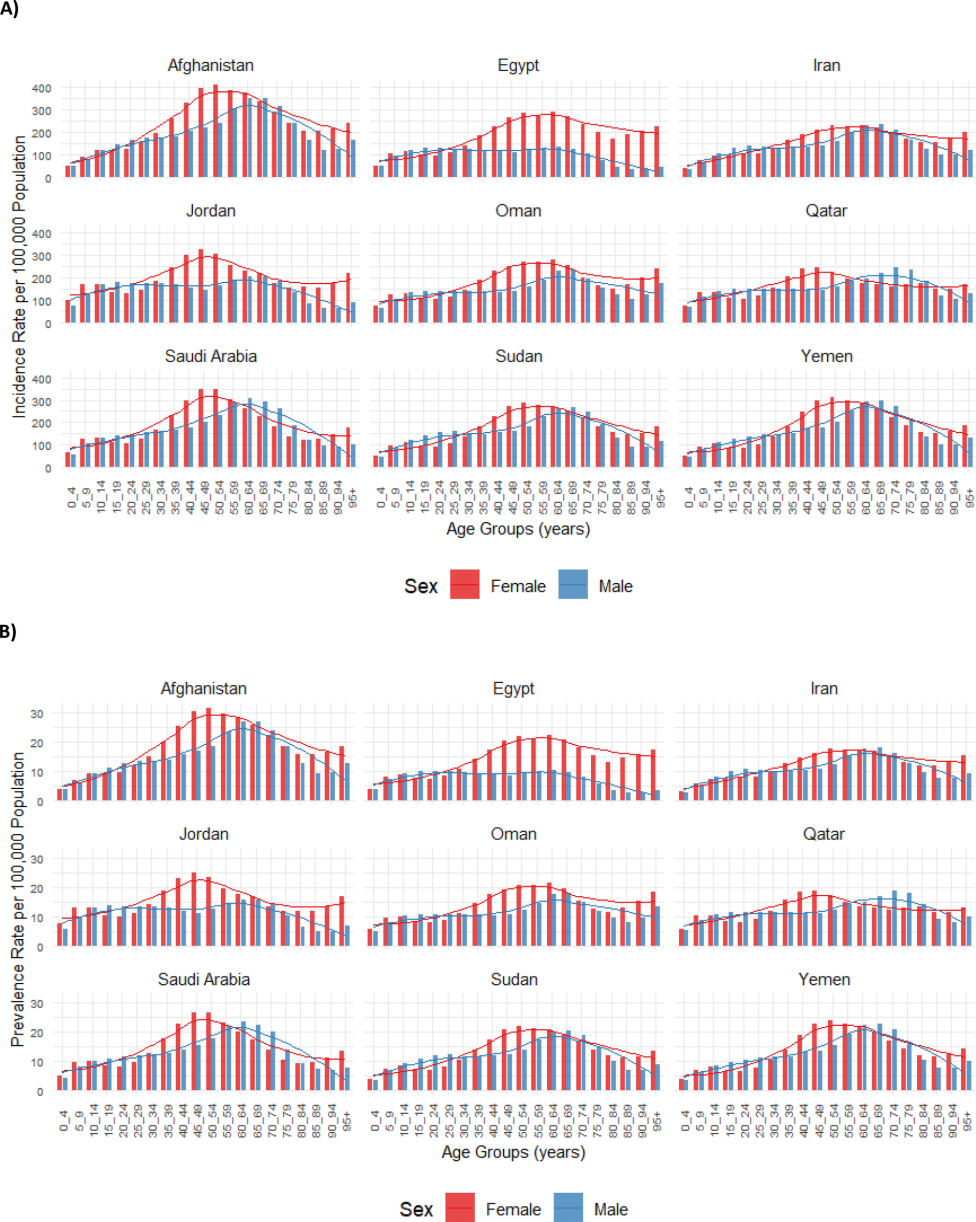

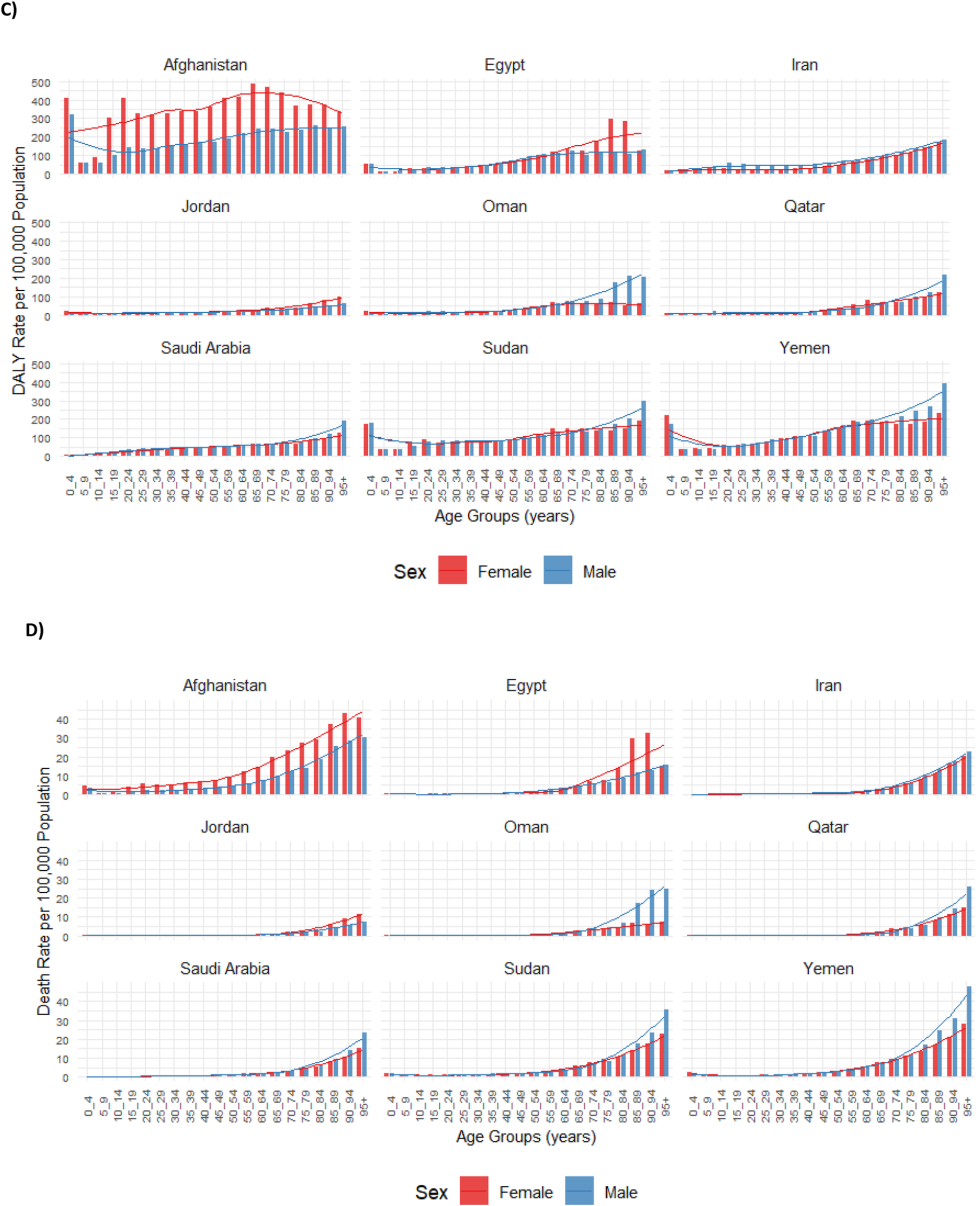
Incidence rate (**A**), prevalence rate (**B**), disability-adjusted life years (DALY) rate (**C**), and death rate (**D**) for adverse effects of medical treatment (AEMT) per 100,000 population in selected countries of the Eastern Mediterranean Region (EMR) in 2021, categorized by location, age, and sex. DALY: disability-adjusted life year. (Generated from data available from http://ghdx.healthdata.org/gbd-results-tool).

In most countries, incidence and prevalence rates rose with age, being the largest in middle-aged groups, followed by a plateau or slight decrease before increasing again in the elderly. Females generally exhibited higher rates, particularly in middle-aged and elderly groups (Fig. 4A and B). Similarly, DALY and death rates increased with age for both sexes, with the burden of AEMT becoming particularly significant in early childhood and older populations for DALY and among elderly individuals for death. In many countries, males and females followed similar patterns across age groups, with sex differences becoming more evident in the elderly (Fig. 4C and D).

In 2021, age-standardized incidence rates of AEMT were higher among females globally, regionally in the EMR, and across all EMR countries, with Afghan females having the highest age-standardized incidence rate among all locations and sexes (Figure S2). The largest decrease in incidence rates from 1990 to 2021 was observed in Iranian males (Figure S3). Similarly, in 2021, females had higher age-standardized point prevalence globally, in the EMR, and across all 22 EMR countries. Afghan females had the highest age-standardized point prevalence among all locations and sexes (Figure S4). Iranian males experienced the largest decrease in age-standardized point prevalence of AEMT from 1990 to 2021 (Figure S5).

Globally and in the EMR, females had higher age-standardized DALY rates in 2021. Among the 22 EMR countries, Afghan females had the highest age-standardized DALY rate (Figure S6). The greatest differences in percentage change of DALY rates between males and females from 1990 to 2021 were recorded in the UAE and Egypt, where males in the UAE and females in Egypt experienced larger decreases compared to their opposite sex (Figure S7).

In 2021, the age-standardized death rate was higher among males globally, while females had higher rates in the EMR. Among females, the highest age-standardized death rates were observed in Afghanistan, the UAE, and Pakistan (Figure S8). Differences in the percentage change of death rates from 1990 to 2021 were noted between males in the UAE and females in Egypt, with larger decreases than the opposite sex (Figure S9).

### Associations between SDI and incidence and DALY rates of AEMT

**Fig. 5:**
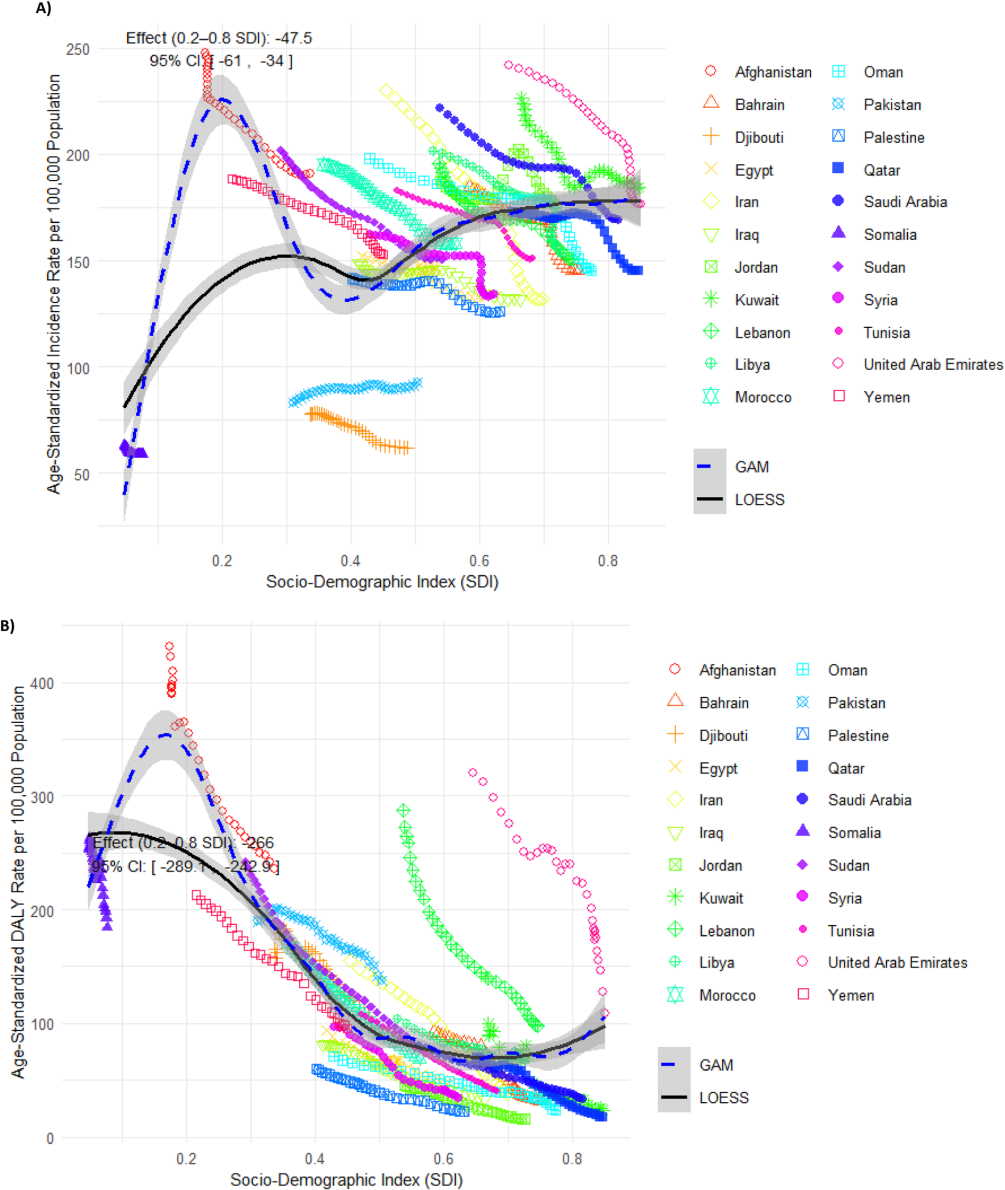
Age-standardized incidence rates (**A**) and age-standardized DALY rates (**B**) of adverse effects of medical treatments (AEMT) per 100,000 population for countries in the Eastern Mediterranean Region (EMR), categorized by SDI from 1990 to 2021. The black line represents the expected values based on SDI and rates across all locations, with the grey area indicating 95% confidence intervals from spline regression. The blue line represents the GAM fit, complementing the LOESS fit shown by the black line. Each point indicates each country’s age-standardized rate per 100,000 population from 1990 to 2021. DALY: disability-adjusted life year. SDI: socio-demographic index. GAM: generalized additive model. LOESS: locally estimated scatterplot smoothing. (Generated from data available from http://ghdx.healthdata.org/gbd-results-tool).

We observed a general positive association between the SDI and the age-standardized incidence rate of AEMT among EMR countries, indicating that as SDI increased, the expected age-standardized incidence rate tended to rise to an SDI of 0.3, followed by a slight decline and then an increase again at higher SDI levels. Given their SDI level, countries such as Afghanistan, Kuwait, and Yemen had a higher age-standardized incidence rate than expected. On the other hand, countries like Pakistan and Djibouti had lower-than-expected age-standardized incidence rates for their given SDI (Fig. 5A). There was a predicted difference in incidence across the SDI range of 0.2 to 0.8 of − 47.5, with estimates from alternative ranges and model settings varying from 9.4 to 54.0 (Figure S10A and Table S3).

There was an overall negative association between SDI and the age-standardized DALY rates due to AEMT among EMR countries (Fig. 5B). Countries such as Afghanistan, Lebanon, and the UAE had higher age-standardized DALY rates than expected, given their SDI. In contrast, countries such as Palestine, Jordan, and Oman showed lower-than-expected age-standardized DALY rates based on their level of development (Fig. 5B). Effect estimates for the SDI and DALY association ranged from − 266.0 to -136.0 across models, consistently indicating a negative association. Sensitivity analysis using narrower SDI ranges and reduced model flexibility confirmed the consistency of these directional associations (Figure S10B and Table S3).

## Discussion

This study examined the epidemiology and burden of AEMT across ages, sexes, and SDI within the EMR at both regional and country levels from 1990 to 2021. The main findings of this study indicated that the incidence, prevalence, DALY and death rates of AEMT were notably high in the EMR, with a decline observed from 1990 to 2021. In 2021, females, early childhood populations, and the elderly generally experienced higher DALY and death rates due to AEMT in the EMR.

Findings indicated that while the EMR had a lower age-standardized rate of incidence and prevalence of AEMT than the global average, it had a higher DALY and death rates due to AEMT. Despite a general decline in AEMT burden in the EMR from 1990 to 2021, AEMT remains a significant healthcare challenge, particularly in developing countries, where socioeconomic and healthcare quality disparities exist^7^. In 2021, DALY and death rates within the EMR ranged from 15.8 to 235.6 and 0.4 to 5.5 per 100,000, respectively, with Afghanistan experiencing the highest rates of AEMT incidence, DALY and death. Contributing factors likely include income level, education, and quality of the healthcare system. Most DALYs and deaths associated with AEMT are concentrated in low and middle-income countries. Among the 22 countries in the EMR, Iran and Kuwait showed the largest decreases in AEMT incidence and death rates from 1990 to 2021, respectively. This decline in age-standardized incidence rates of AEMT in Iran might be attributed to overall healthcare access and quality improvements. However, a systematic review of medical errors in Iran suggested that the lower reported occurrence rate may be due to underreporting, highlighting the need for improvements in AEMT reporting practices^19^. In general, enhancing healthcare staff training, promoting a culture of patient safety, and developing standardized AEMT reporting practices could reduce medical errors and improve healthcare quality in diverse settings^20,21^.

The significant variations across the AEMT burden in the EMR countries reflect greater variation in healthcare systems’ performance, safety culture, and policy implementation. In certain EMR countries, promising interventions have already been implemented to improve patient safety. The WHO Patient Safety Friendly Hospital Initiative (PSFHI) programs were introduced in several countries across the region. Among them, Oman had the most successful pilot, which was implemented in both government and private hospitals. PSFHI addresses patient safety across five key domains: leadership and management, patient and public involvement, safe evidence-based clinical practices, a safe environment, and continuous learning^22^. Qatar is another country in the EMR that has demonstrated a significant commitment to patient safety through creating a national strategy, significant government investment, collaboration with international organizations, and meeting international safety standards. Advanced pharmacovigilance practices in Qatar include a centralized electronic reporting system, compulsory education for health professionals, and regular medication safety recommendations^23^. Iran has also implemented national patient safety plans in collaboration with the WHO and introduced mandatory patient safety standards within its national hospital accreditation program^24^. On the other hand, challenges such as limited resources, political instability, ineffective leadership, absence of national-level regulations, and inadequate access to major healthcare services indicate differences between different jurisdictions in the EMR^25^. In settings of extreme adversity, such as Syria, Yemen, Iraq, Libya, Palestine, Afghanistan, and, indirectly, Lebanon, where countries are experiencing various degrees of emergencies, the primary focus of healthcare systems shifts to saving lives rather than ensuring the quality of care and patient safety^4^.

There was a notable sex-related disparity in incidence, prevalence, DALYs, and deaths due to AEMT, both regionally and in most countries within the EMR. Females may have been more susceptible to AEMT, had a higher likelihood of surgical complications, and experienced medical errors more frequently. Sex disparities in adverse drug events are related to various biological, psychological, and social factors, including differences in the pharmacokinetics and pharmacodynamics of drugs, hormonal variations, pregnancy, and sex-based differences in AEMT reporting^26–28^.

We observed that middle-aged adults in most EMR countries had higher incidence and prevalence rates of AEMT. However, GBD 2019 data indicated that the AEMT incidence rate was higher among older adults globally^2^. A retrospective review of medical records found that the risk of AEMT increased with the number of comorbidities and use of medical devices rather than being intrinsic to age^29^. The occurrence of medical errors among working-age adults could impact their productivity and indirectly lead to an economic burden on individuals and society.

In the EMR, early childhood (0–4 years) and elderly adults (65 + years) experienced a higher burden of DALYs and deaths due to AEMT. Global age-specific death rates from GBD 2017 data demonstrated a bimodal increase in the 0–1 year age group and among those 65 years and older^30^. Due to AEMT, DALY was highest across all WHO regions in the first years of life^5^. These age groups are particularly vulnerable to severe outcomes from AEMT. Younger children and infants are more susceptible to medical errors due to their unique physiological and developmental characteristics^31^. Additionally, children are at higher risk for medication errors due to pharmacological factors, the risk of overdosing, and the need for precise dose calculations^32^. A systematic review by Alsabri et al. indicated that most reported medication errors in pediatric emergency departments are related to dosing errors. They recommended interventions such as electronic medical alert systems, structured ordering systems, accurate weight assessment for pediatric patients, and healthcare professional training in weight-based dosage calculations as effective strategies to reduce medication errors^33^. In older adults, Long et al. highlighted factors such as clinical complexity, comorbidities, reduced functional ability, and lower quality of care contributing to a higher incidence of adverse medical events. Such adverse events often lead to unnecessary interventions, complications, and extended hospital stays for older patients^34^. Recommendations to reduce medical errors in the elderly include establishing dedicated geriatric units in clinical settings, enhancing continuity of care to improve healthcare professionals’ access to patient medical information, and reducing adverse drug events^35^. Additional strategies to minimize medication errors and AEMT in the elderly involve implementing computerized physician order entry, forming committees to review reported errors and determine causality, and double-checking orders by nurses or pharmacists^36^.

We found a positive association between the age-standardized incidence rate of AEMT and the socio-demographic conditions of countries. Generally, the age-standardized incidence rate of AEMT increased with SDI, and countries with relatively higher SDI were expected to have higher incidence rates of AEMT. The GBD 2019 findings indicated that the high SDI quintile had significantly higher and increasing incidence rates compared to lower SDI regions^2^. However, our findings showed a more complex relationship, suggesting that factors beyond SDI, such as differences in healthcare access, AEMT reporting systems, and variations in healthcare policies, might influence the incidence rates of AEMT across EMR countries. Potential residual confounding and reverse causality should be considered when interpreting these associations. Additionally, we observed a negative association between SDI and the age-standardized DALY rate of AEMT, indicating that countries with higher SDI, benefiting from better socioeconomic and healthcare conditions, experience lower DALY rates in the EMR. Prior GBD 2019 findings showed that death and DALY rates of AEMT were lower in countries with higher SDI and are expected to continue decreasing globally^3^. This suggests that while high-SDI countries experienced more AEMT incidents, they managed these events more effectively. This could be related to multiple factors, including developed countries’ patient safety culture, standardized medical error reporting systems, and better healthcare infrastructure to address and manage severe AEMT. A review of safety culture in developing countries reported that patient safety culture and practices are generally lower than those in developed regions^37^. By contrast, developed countries have systematic methods to detect, document, and track medical errors, facilitating learning from errors and helping reduce the incidence and harmful effects of AEMT^38^.

While the region has progressed in patient safety, our findings reveal persistent deficits in healthcare policy and system performance in the EMR countries. The considerable disparities in the AEMT burden, and especially the higher DALY and death rates in lower SDI countries, underscore the urgent need for more comprehensive and context-specific interventions. Despite promising initiatives like the PSFHI in Oman and pharmacovigilance systems in Qatar, many countries still lack standardized AEMT reporting mechanisms, national patient safety strategies, and sustained investment in healthcare quality. Internationally, systems such as the UK’s National Reporting and Learning System (NRLS) and the US Agency for Healthcare Research and Quality (AHRQ) offer important lessons^39,40^. These systems have emphasized non-punitive reporting, continuous learning, and evidence-based methods for promoting safety improvement. These successful practices can be applied to the EMR setting. Based on these findings, we recommend that EMR countries strengthen their health system infrastructure by stratified policy interventions based on feasibility and tailored to the country’s context. Interventions for middle-income countries with stable situations include 1) developing national patient safety strategies aligned with WHO guidance and local needs, 2) implementing digital, standardized reporting systems to capture AEMT events and support continuous learning, 3) investing in workforce training on safety culture, error prevention, and root cause analysis, 4) enhancing patient engagement and feedback mechanisms, and prioritizing high-risk populations, including children and the elderly, through targeted interventions. Interventions for fragile and conflict-affected countries and countries with limited resources include providing basic patient safety training for the healthcare workforce adapted to resource constraints, 2) developing simple or paper-based reporting mechanisms appropriate to their settings, 3) fostering international collaboration programs supporting the incorporation of patient safety principles, 4) integrating patient safety priorities within emergencies, and promoting safety culture among people. Translating these interventions to the specific challenges and resources of each country, especially those within conflict or resource-limited environments, will be essential to eradicating the burden of AEMT and strengthening the overall healthcare profile of the region.

### Strengths and limitations

Our findings on the epidemiology and burden of AEMT have important implications for enhancing the safety and quality of medical care, particularly in the EMR countries. By utilizing secondary analysis of the latest GBD data, we provided a more comprehensive quantification of the burden of AEMT regionally and at the country level in the EMR. Considering the heterogeneity of education, healthcare systems, and economic levels among the EMR countries, we investigated the association of SDI with the incidence and DALY rates of AEMT. Additionally, our analysis examined the contribution of age and sex to the epidemiology of AEMT.

The research methodology of our study has some limitations that should be considered when interpreting the findings. Many low and middle-income countries in our study lack original data on AEMT, leading to reliance on estimation models rather than reported data. For example, primary data on AEMT were unavailable for nine EMR countries, such as Afghanistan, Somalia, and Yemen, and were only partially available for others. This reliance could contribute to potential bias and higher uncertainty in the results of AEMT epidemiology. Reliance on GBD modelling in countries with limited or no primary data on AEMT may reduce the accuracy of estimates, particularly in fragile or conflict-affected settings. Therefore, strengthening local surveillance and standardized reporting systems across the EMR countries is essential to improve data quality and ensure that future patient safety policies are based on context-specific evidence. Additionally, our study analyzed the overall burden of AEMT. Still, due to limited GBD data on specific types of AEMT, we could not evaluate the burden of different medical errors (e.g., medication errors, postoperative infections, and technical complications) in the EMR countries. Future studies analyzing the burden of various types of AEMT are necessary to address these concerns. Furthermore, AEMT has significant financial burdens on healthcare systems, yet our study did not capture the direct and indirect costs associated with AEMT across the EMR countries. We recommend further investigation into the economic impact of AEMT in this region. Finally, potential residual confounding and reverse causality should be considered when interpreting the association between SDI and AEMT burden, as unmeasured differences may influence the observed relationships in healthcare systems, access, or reporting practices rather than actual causal effects.

## Conclusions

Although the incidence, prevalence, DALY, and death rates due to AEMT have declined from 1990 to 2021 in the EMR, the age-standardized DALY and death rates remain higher compared to global figures, particularly among vulnerable populations, including early childhood, the elderly, and females. These findings highlight the necessity for targeted health policy interventions. Key recommendations can include the implementation of comprehensive training programs, the establishment of robust AEMT reporting systems, the promotion of a safety culture within healthcare settings, and efforts to enhance overall healthcare outcomes. Such measures are essential to prevent the occurrence of AEMT in the EMR countries. Furthermore, future studies are recommended to investigate the burden of various types of medical errors using originally reported data, especially in low and middle-income countries within the EMR.

## Supplementary Information

Below is the link to the electronic supplementary material.

Supplementary Material 1

## Data Availability

The data used for these analyses are all publicly available at https://vizhub.healthdata.org/gbd-results/.
