# Supplementary material for: The epidemiology and socioeconomic associates of adverse effects of medical treatment in the Eastern Mediterranean Region from 1990 to 2021
Source: Sci Rep. 2025 Sep 26;15:33232. doi: 10.1038/s41598-025-18289-z (PMC12474870; doi:10.1038/s41598-025-18289-z)
Supplement: Supplementary file 1 — Supplementary Material 1 [file 41598_2025_18289_MOESM1_ESM.docx]

**Table S1.** The International Classification of Disease (ICD) codes used for adverse effects of medical treatment (AEMT) in the Global Burden of Disease 2021.

| **Cause name** | **ICD-9** | **ICD-10** |
| --- | --- | --- |
| Adverse effects of medical treatment | 244.0-244.1, 244.3, 251.3, 253.7, 279.5-279.53, 331.81, 333.92, 349-349.9, 357.6-359.24, 379.6-379.63, 440.3-440.32, 457.0, 458.2-458.29, 518.6-519.1, 525.6-525.79, 526.62-526.63, 530.86-530.87, 536.4-536.49, 539-539.9, 551.2-551.29, 552.2-552.29, 553.2-553.29, 564.2-564.4, 569.6-569.8, 579.3, 595.82, 596.81-596.83, 598.2, 612-612.1, 779.4-780.66, 995.89, E87.0-E87.99, E93.0-E94.99, V44-V45.89 | D69.5-D69.59, D70.1-D70.2, D75.82, D78-D78.89, D89.81-D89.813, E03.2, E06.4, E09-E09.9, E16.0, E23.1, E24.2, E27.3, E36-E36.8, E66.1, E71.43, E89-E89.9, G21.0-G25.79, G62.0-G97.9, H05.33-H05.339, H05.42-H05.53, H59-H59.89, H91.0-H91.09, H95-H95.9, I95.2-I97.9, J70-J70.4, J95-J95.9, K08.5-K08.59, K43-K43.9, K52.0, K62.7, K68.11, K91-K91.9, K94-K95.89, L23.3, L27.0-L27.1, L56.0-L56.1, L64.0, L76-L76.82, M10.2-M10.29, M87.1-M87.19, M96-M96.9, N14-N14.4, N30.4-N30.41, N46.021-N46.121, N52.2-N52.39, N65-N65.1, N99-N99.9, P93-P93.8, P96.2-P96.5, R50.2-R50.83, Y40-Y84.9, Y88-Y88.3, Z21.0, Z42-Z51.9, Z88-Z94.0, Z94.6-Z99.9 |

**Table S2.** Prevalent cases, incident cases, deaths, and DALY, along with their age-standardized rates, due to adverse effects of medical treatments in 2021 for both sexes and the percentage change in rates per 100,000 population from 1990 to 2021 in the Eastern Mediterranean Region (EMR).

| **Location** | **Incidence (95% UI)** | | |  | **Prevalence (95% UI)** | | |  | **DALY (95% UI)** | | |  | **Deaths (95% UI)** | | |
| --- | --- | --- | --- | --- | --- | --- | --- | --- | --- | --- | --- | --- | --- | --- | --- |
|  | **Counts**  **(2021)** | **Rate**  **(2021)** | **Pcs in rate**  **1990-2021** |  | **Counts**  **(2021)** | **Rate**  **(2021)** | **Pcs in rate**  **1990-2021** |  | **Counts**  **(2021)** | **Rate**  **(2021)** | **Pcs in rate**  **1990-2021** |  | **Counts**  **(2021)** | **Rate**  **(2021)** | **Pcs in rate**  **1990-2021** |
| **Global** | 12481276.3 (10886792.7, 14290629.9) | 150.4 (131.2, 171.8) | -5.3 (-7.9, -2.6) |  | 952439.5 (730685.1, 1180518.8) | 11.5 (8.9, 14.1) | -5.3 (-7.8, -2.7) |  | 4846980.9 (3914845, 5494171.1) | 64.2 (51.1, 73.1) | -39.7 (-48.9, -31.2) |  | 122330.5 (103909.7, 133910.6) | 1.5 (1.3, 1.7) | -36.1 (-43.7, -28) |
| **Eastern Mediterranean Region** | 905995.8 (765960.8, 1077224.5) | 127.9 (110.4, 149.8) | -18.7 (-20.5, -17) |  | 69234.7 (53998.1, 85948.9) | 9.8 (7.7, 12.1) | -18.7 (-20.5, -17) |  | 602326.4 (495340.2, 704858.5) | 88.6 (73.6, 103.2) | -46.3 (-55.4, -35.4) |  | 12644.5 (10534.9, 14722) | 2.4 (2, 2.8) | -40.7 (-54.8, -27.3) |
| **Afghanistan** | 45865.4 (39677.2, 54132.5) | 190.7 (169.5, 217.4) | -22.9 (-28.3, -17.5) |  | 3505 (2724.4, 4292.4) | 14.6 (11.4, 17.6) | -22.9 (-28.4, -17.4) |  | 67239 (44839.9, 93977.2) | 235.6 (160.3, 326.8) | -45.4 (-57.6, -29.3) |  | 1121.8 (758.3, 1579.7) | 5.5 (3.7, 7.8) | -39.3 (-54.3, -19.6) |
| **Bahrain** | 2290.4 (1870.4, 2762.5) | 145.3 (123, 173.7) | -21.4 (-25, -17.4) |  | 175 (133, 226.2) | 11.1 (8.5, 14) | -21.4 (-25.2, -17.4) |  | 403.7 (323.4, 493.8) | 31.2 (25.2, 38.1) | -66.1 (-75.8, -55) |  | 9.3 (7.4, 11.5) | 1.1 (0.9, 1.3) | -62.6 (-73.9, -50.6) |
| **Djibouti** | 706.6 (595.4, 828.3) | 62.1 (53.4, 72.3) | -20.2 (-23.7, -16.2) |  | 54 (41.6, 67.1) | 4.7 (3.7, 5.9) | -20.2 (-23.7, -16.1) |  | 1115.9 (698.6, 2181.6) | 101.2 (62.2, 207.8) | -38.9 (-57.4, -12.1) |  | 21.6 (13, 46.6) | 2.8 (1.7, 6.4) | -24.6 (-44.8, 4.2) |
| **Egypt** | 134543.4 (111507.2, 163920.8) | 133 (112, 160.3) | -12.6 (-16.4, -7.9) |  | 10282 (7891.3, 13053.2) | 10.2 (7.8, 12.8) | -12.6 (-16.4, -8) |  | 44110.2 (32238.8, 54998.6) | 48.1 (35.7, 59.9) | -48.6 (-66.3, -24.7) |  | 989.6 (717.9, 1246.7) | 1.4 (1.1, 1.7) | -40.8 (-62.5, -12.1) |
| **Iran** | 117248.4 (99458.8, 138900.2) | 131.8 (113.1, 155.9) | -42.7 (-44.5, -41) |  | 8959 (6922.7, 11289.7) | 10.1 (7.9, 12.7) | -42.7 (-44.5, -41) |  | 32170.8 (27302.1, 40114.6) | 38.4 (33.1, 47.6) | -75.3 (-83, -64.7) |  | 822.3 (698.8, 1002.4) | 1.1 (0.9, 1.3) | -67.1 (-77.7, -54.2) |
| **Iraq** | 52780.6 (43713.6, 64118.2) | 131.9 (111.3, 157.1) | -11.5 (-15.3, -7.7) |  | 4033.4 (3087.9, 5128.6) | 10.1 (7.8, 12.6) | -11.5 (-15.3, -7.7) |  | 12269.6 (9480.2, 16163.3) | 33.5 (25.5, 43.4) | -58.7 (-72.3, -41.1) |  | 256.8 (190.9, 340.9) | 0.9 (0.7, 1.1) | -50.8 (-70.9, -26.6) |
| **Jordan** | 21064.3 (17322.8, 26376.8) | 171 (143.3, 211) | -8.9 (-14.4, -2.9) |  | 1609.7 (1224.4, 2087.9) | 13.1 (9.9, 16.9) | -8.9 (-14.6, -2.6) |  | 1753 (1424.5, 2131.2) | 15.8 (12.8, 19.2) | -65.4 (-75.1, -53.1) |  | 33.9 (27.1, 42.2) | 0.4 (0.3, 0.5) | -65.4 (-77.8, -50.6) |
| **Kuwait** | 8867.6 (7319.2, 10561.7) | 184.5 (157.8, 216.4) | -18.5 (-22, -15) |  | 677.6 (512.8, 863.4) | 14.1 (10.9, 17.6) | -18.5 (-22, -14.9) |  | 961 (796.3, 1149.3) | 23.8 (19.8, 28.3) | -83.1 (-85.9, -80) |  | 22.2 (18.1, 26.9) | 0.7 (0.6, 0.8) | -80.9 (-84.7, -76.9) |
| **Lebanon** | 8568.8 (7222, 10130.9) | 150.6 (126.9, 180.3) | -23.1 (-27.1, -18.6) |  | 654.7 (505.5, 819.9) | 11.5 (9, 14.4) | -23.1 (-27.2, -18.6) |  | 5629.1 (4694.7, 6829.2) | 97.3 (81.4, 118.1) | -66.1 (-73.1, -55.6) |  | 184.2 (149, 220.6) | 3 (2.4, 3.6) | -61.1 (-70.1, -47.7) |
| **Libya** | 10953.8 (9249.8, 12958.7) | 153.4 (130.8, 178.5) | -23.9 (-27.3, -19.9) |  | 837 (647.9, 1040) | 11.7 (9.2, 14.6) | -23.9 (-27.3, -20) |  | 4453.6 (3166.1, 6074.8) | 67 (47.7, 89.9) | -35.6 (-55.5, -11.2) |  | 103.2 (72.6, 148.3) | 1.8 (1.2, 2.6) | -28.7 (-50.3, -2.3) |
| **Morocco** | 59937.8 (51793.8, 69630.9) | 158.2 (136.6, 183) | -19 (-23.7, -14.1) |  | 4579.5 (3586, 5688.5) | 12.1 (9.5, 14.9) | -19 (-23.7, -14.2) |  | 25616.8 (18016.4, 37201.3) | 70.2 (49.7, 100.6) | -58.9 (-68.6, -45.6) |  | 642.3 (458.8, 871.3) | 1.9 (1.4, 2.5) | -46.9 (-61.2, -28.2) |
| **Oman** | 6602.9 (5321.3, 7977.9) | 145.4 (121.9, 173.3) | -26.6 (-31.4, -21.8) |  | 504.7 (379.4, 643.8) | 11.1 (8.6, 14) | -26.6 (-31.5, -21.9) |  | 876.9 (686.9, 1071) | 23.2 (18.2, 27.9) | -67.5 (-81.1, -50) |  | 18.2 (13.9, 22.4) | 0.7 (0.5, 0.9) | -61.3 (-78.7, -38.6) |
| **Pakistan** | 191708.3 (163134.5, 224386.8) | 92.7 (80.4, 107.3) | 11.8 (6, 16.9) |  | 14651.2 (11341.1, 18126.3) | 7.1 (5.5, 8.8) | 11.8 (5.8, 17.2) |  | 270071.1 (218249.6, 325048.7) | 137.5 (113.8, 165.9) | -27.5 (-46.8, -7.4) |  | 5929.2 (4913.9, 7207.1) | 4.3 (3.5, 5.3) | -19.6 (-44.8, 6.9) |
| **Palestine** | 6113.4 (5044.2, 7542.8) | 126 (106, 153) | -11.1 (-14.9, -7.7) |  | 467.2 (355.8, 605) | 9.6 (7.5, 12.3) | -11.1 (-14.7, -7.7) |  | 954.7 (802, 1178.8) | 22.4 (18.9, 27.4) | -62.9 (-74.5, -48.1) |  | 19.7 (16.5, 24.6) | 0.6 (0.5, 0.8) | -59.2 (-74.7, -40.1) |
| **Qatar** | 4395 (3448.3, 5486.7) | 145.1 (121.3, 176.5) | -16 (-20.2, -11.6) |  | 336 (244.2, 442.7) | 11.1 (8.4, 14.2) | -16 (-20.3, -11.7) |  | 400.7 (306.5, 521.3) | 17.3 (13.8, 21.7) | -75.1 (-83.4, -62) |  | 7.6 (5.6, 10.1) | 0.6 (0.4, 0.7) | -75.1 (-85, -60.7) |
| **Saudi Arabia** | 66060.1 (55223.5, 78004.2) | 169.3 (145.3, 201) | -23.6 (-29, -18.8) |  | 5049 (3851.9, 6389.1) | 12.9 (10, 16.1) | -23.6 (-29.2, -18.9) |  | 13117.1 (9016.2, 18126.6) | 33.7 (25.2, 44.2) | -60.1 (-75, -37.5) |  | 270.8 (186.7, 373.3) | 0.9 (0.7, 1.2) | -55.2 (-72.8, -30.6) |
| **Somalia** | 9813.7 (8321.7, 11518.7) | 58.9 (50.9, 67.8) | -6.6 (-10.9, -1.4) |  | 750 (583.2, 929.4) | 4.5 (3.5, 5.5) | -6.6 (-10.9, -1.4) |  | 40236.7 (21976.4, 101491.3) | 184.2 (96.9, 583.3) | -27.2 (-50.1, 2.6) |  | 617.9 (334.4, 1768.4) | 4.8 (2.4, 18.2) | -17.2 (-40.4, 6.2) |
| **Sudan** | 57536.6 (48175.4, 68998.5) | 150.7 (129.5, 174.3) | -25.5 (-29.3, -20.6) |  | 4396.4 (3391.3, 5495.7) | 11.5 (9, 14.3) | -25.5 (-29.4, -20.4) |  | 36188.9 (23394.6, 51844.8) | 88.7 (58, 126.3) | -63.4 (-74.5, -48.7) |  | 642.3 (419.1, 917.2) | 2.1 (1.4, 2.9) | -56.7 (-70.2, -38.7) |
| **Syria** | 19487.3 (16584.2, 23459.3) | 133.8 (112.9, 159.9) | -17.5 (-22.6, -13.3) |  | 1488.8 (1160.7, 1884) | 10.2 (7.9, 12.8) | -17.5 (-22.5, -13.3) |  | 4721.5 (3172.3, 6367.3) | 34.6 (23.2, 46.4) | -64.4 (-79, -43.8) |  | 124.7 (82.2, 167.7) | 1 (0.7, 1.4) | -56.6 (-75.7, -30) |
| **Tunisia** | 18938.5 (16217.4, 22419.8) | 151 (128.6, 180.8) | -17.3 (-21.1, -13.2) |  | 1447 (1112.5, 1814.9) | 11.5 (8.9, 14.5) | -17.3 (-21, -13.2) |  | 4852.8 (3554, 6455.1) | 39.8 (29.2, 51.9) | -63 (-76.6, -47.9) |  | 134.7 (94.5, 183.7) | 1.1 (0.8, 1.5) | -54 (-73, -33.2) |
| **United Arab Emirate** | 19284 (15698.3, 23028.9) | 176.8 (150.9, 207) | -27 (-30.4, -23.7) |  | 1473.9 (1094.1, 1880.9) | 13.5 (10.4, 16.8) | -27 (-30.3, -23.5) |  | 6823.8 (5103.6, 9135.9) | 108.3 (85.9, 132.2) | -66.2 (-77.9, -52.3) |  | 151 (113.5, 202.1) | 3.8 (3, 4.6) | -59.1 (-73, -41.8) |
| **Yemen** | 43228.9 (36597.7, 51467.8) | 152.9 (133.9, 174.4) | -18.9 (-23.7, -13.4) |  | 3303.4 (2565.2, 4109.7) | 11.7 (9.1, 14.2) | -18.9 (-23.7, -13.5) |  | 28359.4 (18408.9, 41225.3) | 95.5 (61.9, 138.7) | -55.2 (-66.9, -37.7) |  | 521.3 (335.4, 763.5) | 2.4 (1.6, 3.6) | -46.9 (-62.5, -23.7) |

**Abbreviations:** DALY: disability-adjusted life year; Pcs: percent changes; UI: uncertainty interval.

**Table S3**. Effect estimates from GAM spline models across SDI ranges and smoothing parameters for incidence and DALY outcomes.

| **Outcome** | **SDI range** | **Model** | **Effect Estimate** |
| --- | --- | --- | --- |
| Incidence | 0.2-0.8 | GAM (default) | -47.54 |
| Incidence | 0.3-0.7 | GAM (default) | 9.41 |
| Incidence | 0.2-0.8 | GAM (k=4) | 54.04 |
| DALY | 0.2-0.8 | GAM (default) | -266.04 |
| DALY | 0.3-0.7 | GAM (default) | -136.04 |
| DALY | 0.2-0.8 | GAM (k=4) | -150.76 |

A)


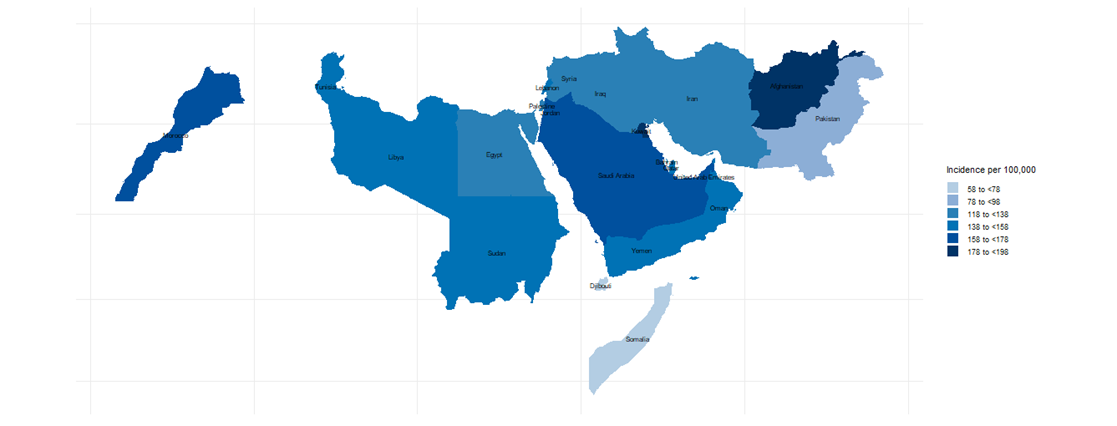


B)


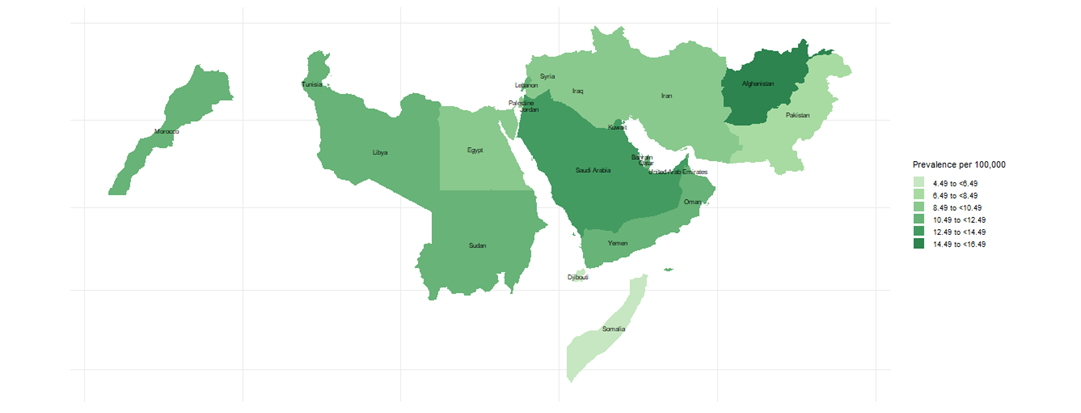


C)


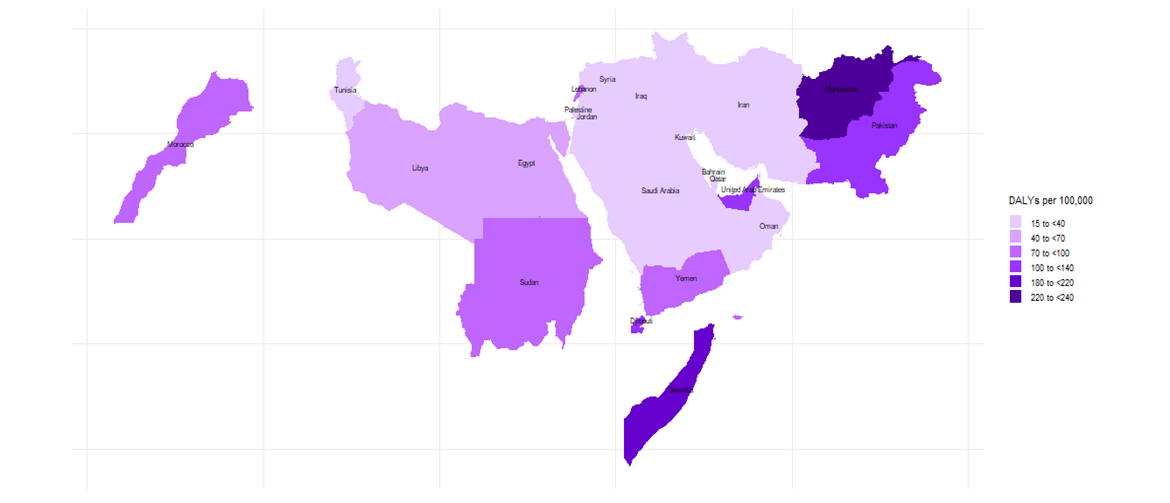


D)


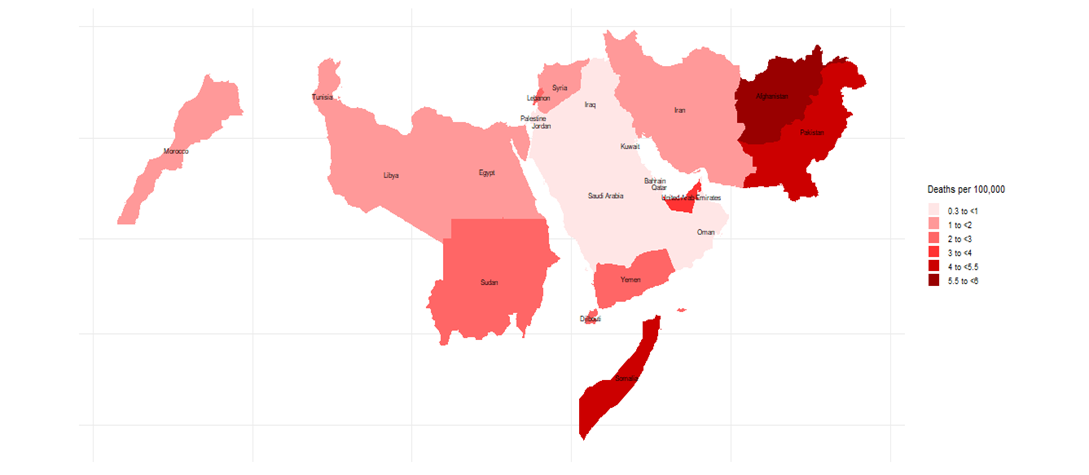


**Figure S1.** Age-standardized rates of incidence (A), prevalence (B), disability-adjusted life years (DALYs) (C), and deaths (D) of adverse effects of medical treatment (AEMT) (per 100,000 population) in 2021 for countries in the Eastern Mediterranean Region (EMR). (Generated from data available from <http://ghdx.healthdata.org/gbd-results-tool>).


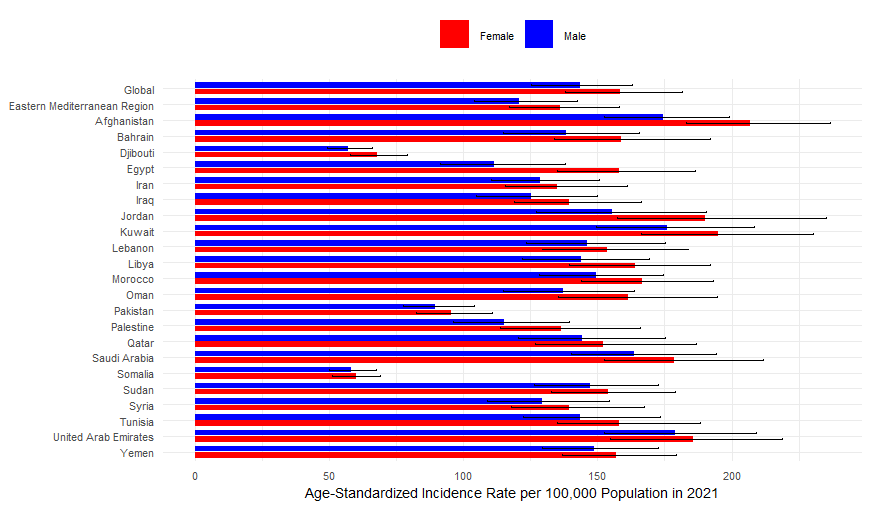


**Figure S2.** Age-standardized incidence rates of adverse effects of medical treatment (AEMT) per 100,000 population in 2021, by location and sex. (Generated from data available at <http://ghdx.healthdata.org/gbd-results-tool>)


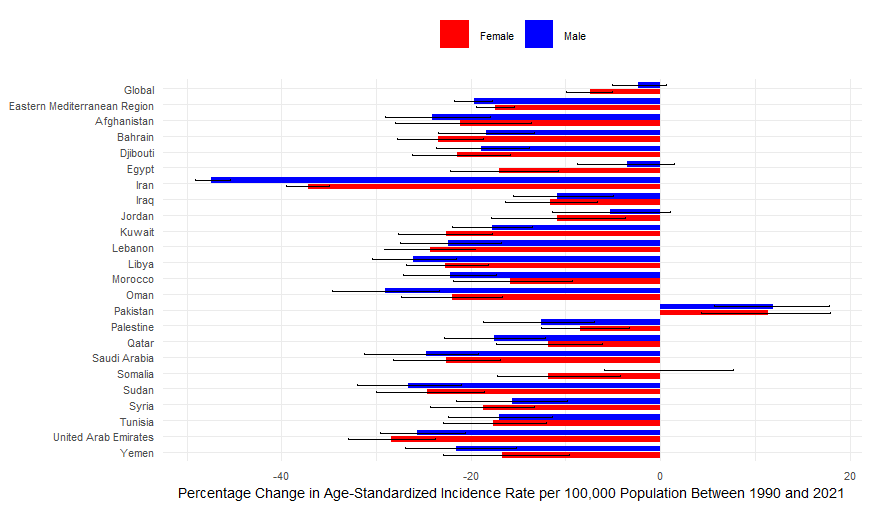


**Figure S3.** Percentage change in the age-standardized incidence rates of adverse effects of medical treatment (AEMT) from 1990 to 2021, by location and sex. (Generated from data available at <http://ghdx.healthdata.org/gbd-results-tool>)


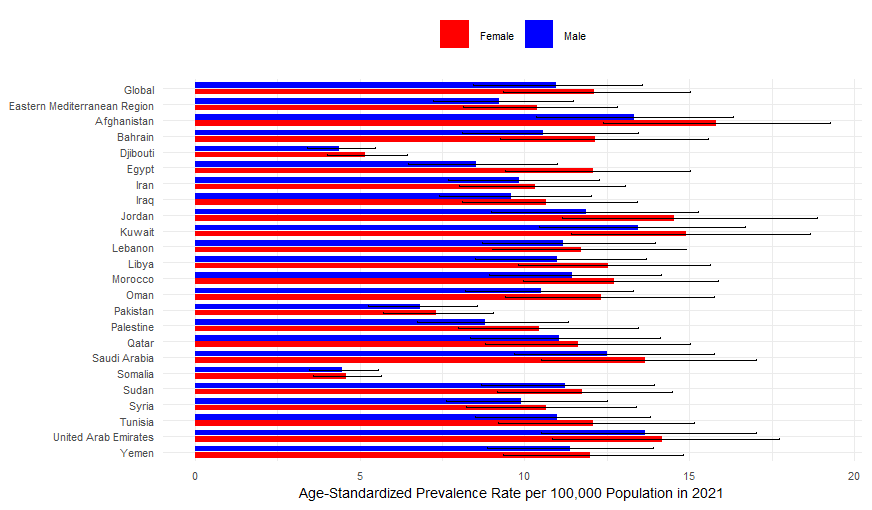


**Figure S4.** Age-standardized prevalence rates of adverse effects of medical treatment (AEMT) per 100,000 population in 2021, by location and sex. (Generated from data available at <http://ghdx.healthdata.org/gbd-results-tool>).


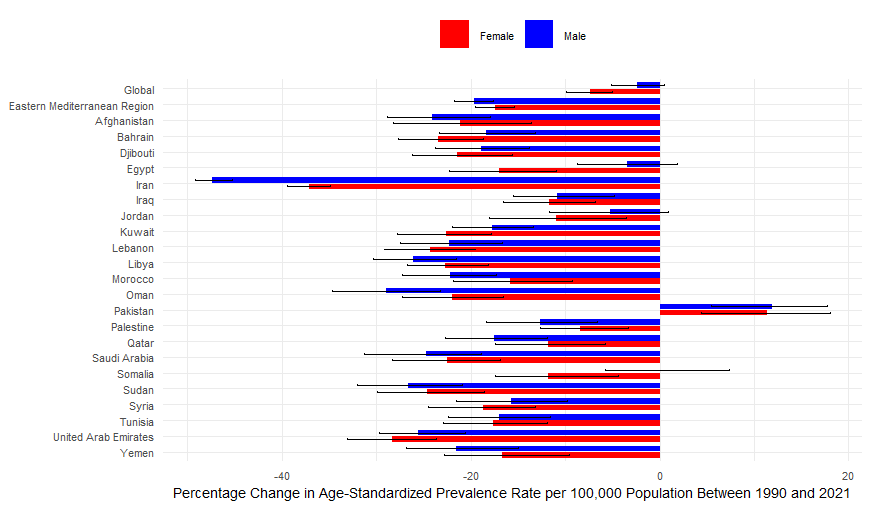


**Figure S5.** The percentage change in age-standardized prevalence rates of adverse effects of medical treatment (AEMT) from 1990 to 2021, by location and sex. (Generated from data available at <http://ghdx.healthdata.org/gbd-results-tool>).

**
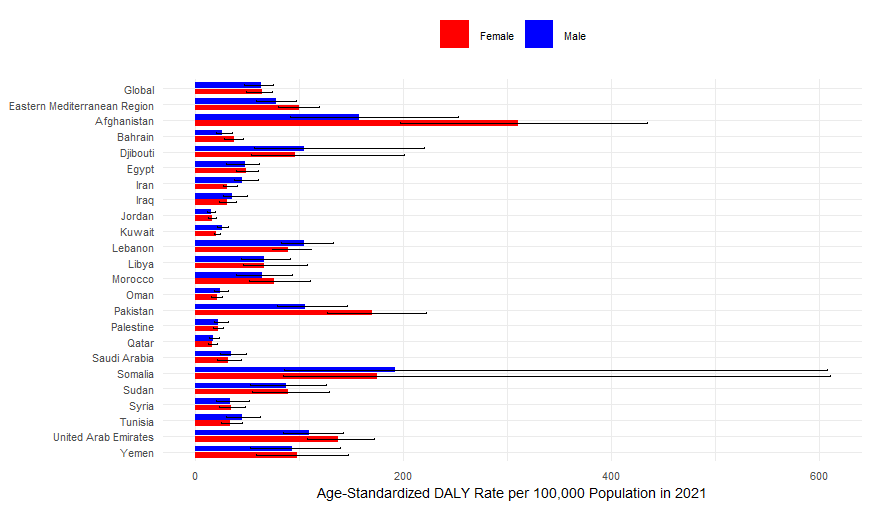
**

**Figure S6**. Age-standardized disability-adjusted life year (DALY) rates for adverse effects of medical treatment (AEMT) per 100,000 population in 2021, by location and sex. DALY= disability-adjusted life year. (Generated from data available at <http://ghdx.healthdata.org/gbd-results-tool>).


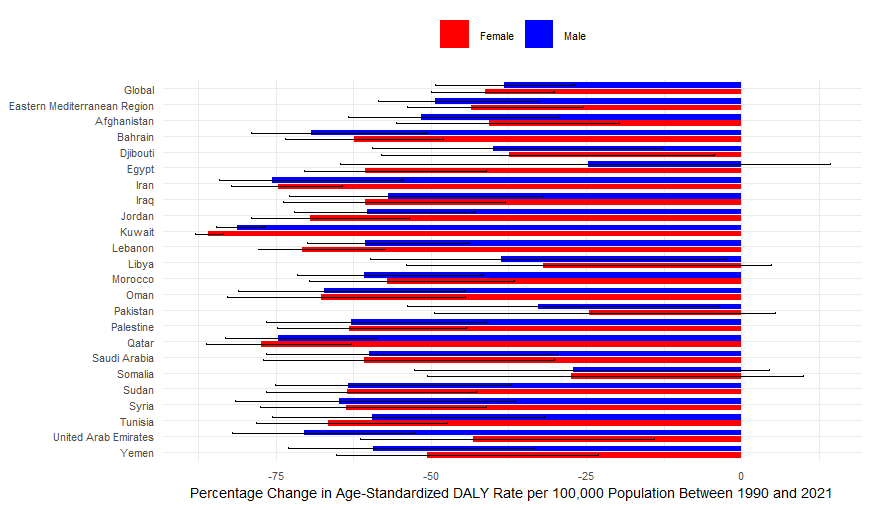


**Figure S7**. Percentage change in the age-standardized disability-adjusted life year (DALY) rates of adverse effects of medical treatment (AEMT) from 1990 to 2021, by location and sex. (Generated from data available at <http://ghdx.healthdata.org/gbd-results-tool>).


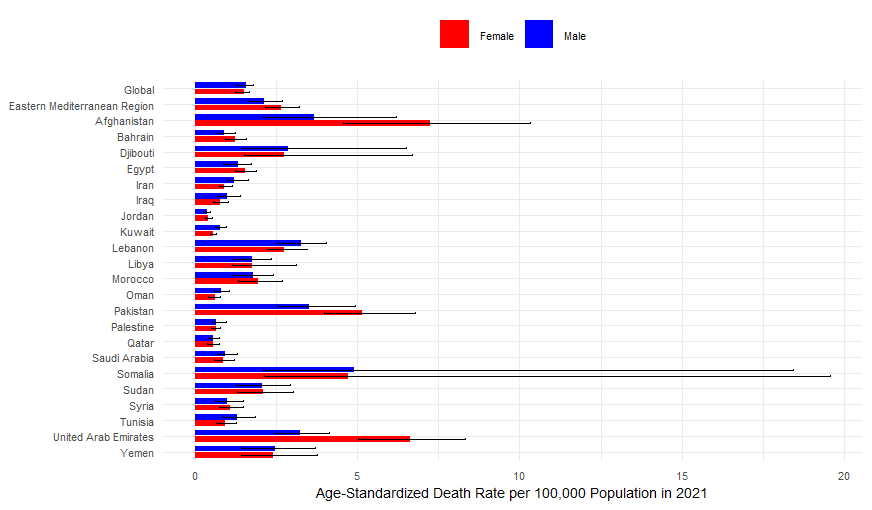


**Figure S8**. Age-standardized death rates for adverse effects of medical treatment (AEMT) per 100,000 population in 2021, by location and sex. (Generated from data available at <http://ghdx.healthdata.org/gbd-results-tool>).


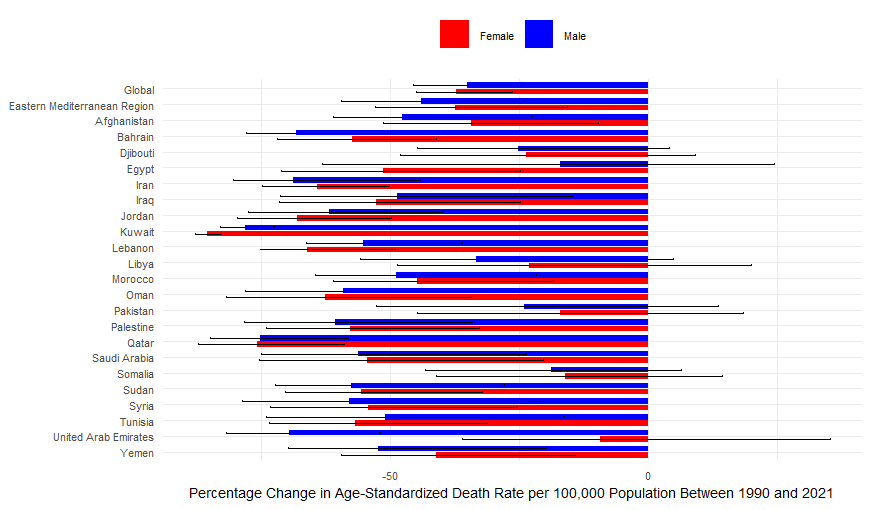


**Figure S9**. Percentage change in the age-standardized death rate of adverse effects of medical treatment (AEMT) from 1990 to 2021, by location and sex. (Generated from data available at <http://ghdx.healthdata.org/gbd-results-tool>)

**A**


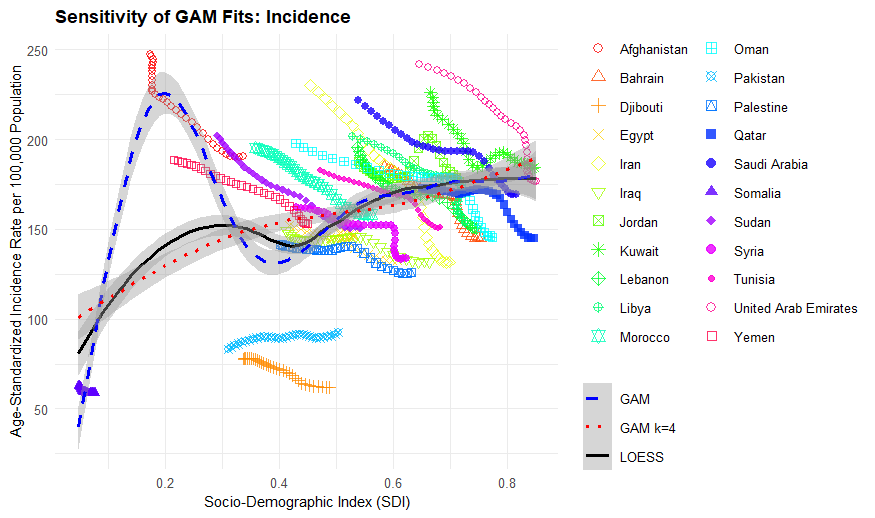


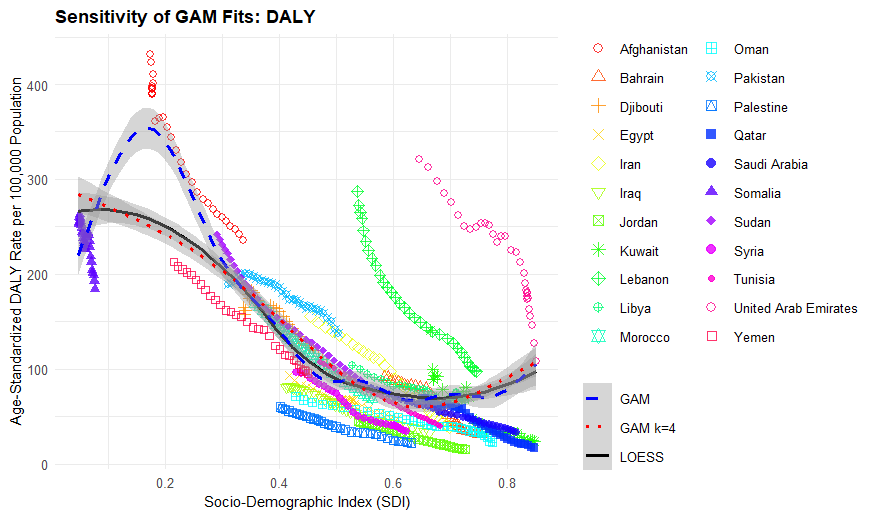


**Figure S10**. Sensitivity analysis of generalized additive model (GAM) spline fits for the association between Socio-demographic Index (SDI) and age-standardized rates of (A) incidence and (B) DALY due to adverse events of medical treatment (AEMT). The LOESS smoother (solid black line), default GAM (blue dashed line), and GAM with reduced degrees of freedom (red dotted line, k = 4) are shown. Confidence intervals (95%) are shaded. This sensitivity check supports the robustness of the nonlinear trends to spline specification.
